# Paediatric genomic testing: Navigating medicare rebatable genomic testing

**DOI:** 10.1111/jpc.15382

**Published:** 2021-02-10

**Authors:** Rani Sachdev, Mike Field, Gareth S Baynam, John Beilby, Maria Berarducci, Yemima Berman, Tiffany Boughtwood, Marie B Cusack, Vanessa Fitzgerald, Jeffery Fletcher, Mary‐Louise Freckmann, Natalie Grainger, Edwin Kirk, Ben Lundie, Sebastian Lunke, Lesley McGregor, David Mowat, Gayathri Parasivam, Vanessa Tyrell, Mathew Wallis, Susan M White, Alan S L Ma

**Affiliations:** ^1^ Centre for Clinical Genetics, Sydney Children's Hospital‐Randwick Sydney Children's Hospitals Network Sydney New South Wales Australia; ^2^ School of Women's and Children's Health University of New South Wales Sydney New South Wales Australia; ^3^ Cancer Genetics Royal North Shore Hospital Sydney New South Wales Australia; ^4^ GOLD Service Hunter‐New England Health Service Newcastle New South Wales Australia; ^5^ Department of Health Genetic Services of Western Australia Perth Western Australia Australia; ^6^ Department of Diagnostic Genomics PathWest Laboratory Medicine Perth Western Australia Australia; ^7^ Health Education and Training Institute (HETI) NSW Health Service Sydney New South Wales Australia; ^8^ Department of Clinical Genetics Royal North Shore Hospital Sydney New South Wales Australia; ^9^ Sydney Medical School University of Sydney Sydney New South Wales Australia; ^10^ Australian Genomics Parkville Victoria Australia; ^11^ Murdoch Children's Research Institute Parkville Victoria Australia; ^12^ NSW Health Centre for Genetics Education Royal North Shore Hospital Sydney New South Wales Australia; ^13^ Speciality Services and Technology Evaluation Unit, Strategic Reform and Planning Branch NSW Ministry of Health Sydney New South Wales Australia; ^14^ Department of Paediatrics The Tweed Hospital Tweed Heads New South Wales Australia; ^15^ Randwick Genomics Laboratory NSW Health Pathology Sydney New South Wales Australia; ^16^ Pathology Queensland Royal Brisbane and Women's Hospital Brisbane Queensland Australia; ^17^ Victorian Clinical Genetics Services Murdoch Children's Research Institute Melbourne Victoria Australia; ^18^ Department of Pathology University of Melbourne Melbourne Victoria Australia; ^19^ South Australian Clinical Genetics Service Women's and Children's Hospital Adelaide South Australia Australia; ^20^ Children's Cancer Institute. Randwick Sydney New South Wales Australia; ^21^ Tasmanian Clinical Genetics Service, Tasmanian Health Service Royal Hobart Hospital Hobart Tasmania Australia; ^22^ School of Medicine The University of Tasmania Hobart Tasmania Australia; ^23^ Department of Paediatrics University of Melbourne Melbourne Victoria Australia; ^24^ Specialty of Genomic Medicine University of Sydney Sydney New South Wales Australia; ^25^ Department of Clinical Genetics, Children's Hospital Westmead Sydney Children's Hospitals Network Sydney New South Wales Australia

## Abstract

Genomic testing for a genetic diagnosis is becoming standard of care for many children, especially those with a syndromal intellectual disability. While previously this type of specialised testing was performed mainly by clinical genetics teams, it is increasingly being ‘mainstreamed’ into standard paediatric care. With the introduction of a new Medicare rebate for genomic testing in May 2020, this type of testing is now available for paediatricians to order, in consultation with clinical genetics. Children must be aged less than 10 years with facial dysmorphism and multiple congenital abnormalities or have global developmental delay or moderate to severe intellectual disability. This rebate should increase the likelihood of a genetic diagnosis, with accompanying benefits for patient management, reproductive planning and diagnostic certainty. Similar to the introduction of chromosomal microarray into mainstream paediatrics, this genomic testing will increase the number of genetic diagnoses, however, will also yield more variants of uncertain significance, incidental findings, and negative results. This paper aims to guide paediatricians through the process of genomic testing, and represents the combined expertise of educators, clinical geneticists, paediatricians and genomic pathologists around Australia. Its purpose is to help paediatricians navigate choosing the right genomic test, consenting patients and understanding the possible outcomes of testing.

Paediatric genetic disease accounts for 2–14% of all hospital admissions^1^ and has a high financial, societal and health‐care burden.^2^ Identifying a genetic diagnosis via genetic or genomic testing (GT) enhances management and, in almost one‐third of cases, directs specific treatment or a change in care.^3^ In addition, it allows recurrence risk estimation, enables the identification of at‐risk family members, shortens the diagnostic odyssey, reduces invasive investigations and provides psychosocial benefits, including closure for families.^4^

GT, which examines the DNA sequence of a patient's genes, is becoming a standard part of clinical practice, and as a result, the number of patients receiving a genetic diagnosis has increased in recent years. This type of testing, once mainly performed in the clinical genetics clinic, is now being ‘mainstreamed’ into general paediatric practice, with the introduction of a Medicare rebate for patients aged 9 years 11 months or less with suspected monogenic conditions (Medicare Benefits Schedule (MBS) item numbers 73358–73361; Fig. 1).^5^

**Fig. 1:**
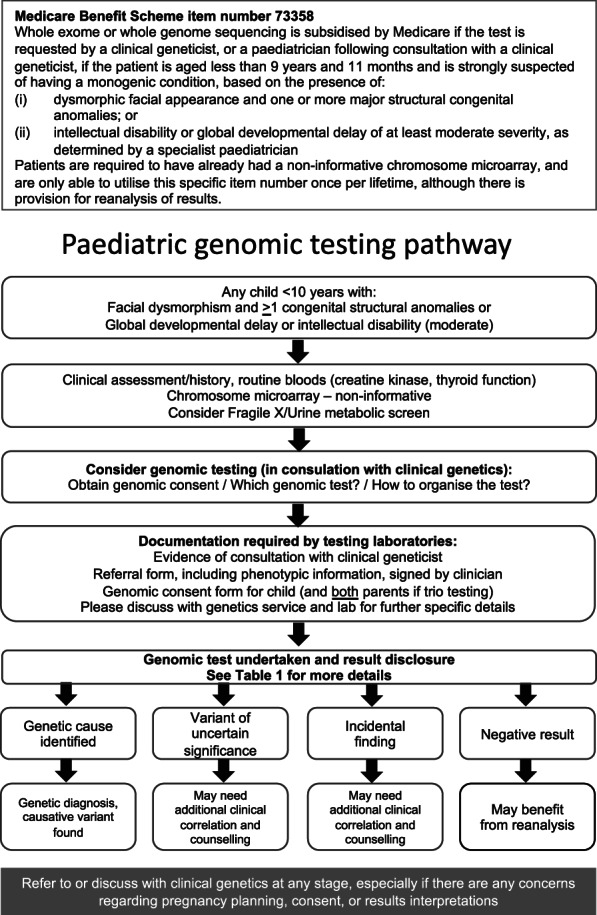
Suggested pathway for genomic testing under Medicare Benefits Schedule item 73358.

Next generation sequencing (NGS), also known as massively parallel sequencing, enables the simultaneous sequencing of millions of bases of DNA. This tool has transformed the diagnostic landscape and identifies the monogenetic aetiologies of neurodevelopmental and multiple congenital anomaly (MCA) syndromes. Current diagnostic yield from GT is 29–57% in paediatric patients suspected of having an underlying monogenetic condition.^6^

With the widespread adoption of NGS, the range of genetic and genomic tests available to clinicians has expanded. This has been accompanied by decreasing cost as more enhanced testing platforms evolve. Historically, access to whole exome sequencing (WES; protein‐coding DNA) and/or whole genome sequencing (WGS; all DNA) has been limited, largely due to cost and the availability of public funding.

With the introduction of this new Medicare item, paediatricians will need to become familiar with the process of GT, including: (i) recognising when and in which patients testing is clinically warranted; (ii) fulfilling pre‐test requirements; (iii) choosing and organising the appropriate test, and obtaining informed consent; and (iv) understanding, interpreting, and explaining results.

We present advice from Australian clinical genetics experts to assist paediatricians in utilising the Medicare items.

### Recognising when GT is indicated

#### Patient selection

Recognising that a patient warrants GT is critical and depends on various factors. Firstly, the Medicare item numbers are only applicable to patients seen in the outpatient setting. Secondly, diagnostic yield is an important consideration and is largely related to the patient's phenotypic presentation (Fig. 1). Specific patient cohorts have a higher likelihood of a monogenic disorder, such as those with dysmorphism, moderate–severe intellectual disability (ID) and MCA.^7^, ^8^ Patients with isolated abnormalities (e.g. single organ involvement such as cleft palate, isolated autism or attention‐deficit/hyperactivity disorder or dysmorphism without significant co‐morbidities, or milder delay, will not qualify for testing, due to low yield and the complex/multifactorial nature of these conditions.^9^, ^10^, ^11^, ^12^ Conversely, GT is indicated in some clinical presentations (e.g. congenital hearing loss, neurofibromatosis type 1) even though these patients do not qualify for the rebate. In these instances, referral to the local genetics service is recommended per usual practice, especially if there are issues such as reproductive planning.

### Requirements before testing can be ordered

#### First‐tier testing

Genomic analysis is an expansive and expensive test; therefore, routine first tier investigations should not be overlooked (Fig. 1). These include clinical assessment, history, and routine blood testing (e.g. electrolytes, full blood count, thyroid function, creatine kinase and liver function tests); chromosomal microarray (CMA) testing (consistent with MBS item 73 292)^5^ must also have been performed and be non‐informative. CMA screens the patient's chromosomes for microdeletions and microduplications, also known as copy number variants (CNVs). These are found in 5–15% of children with ID or global developmental delay (GDD).^13^, ^14^ CNVs cannot currently be detected using WES, making this first step critical to ensure pathogenic CNVs are detected. Ordering CMA is now part of routine paediatric practice and will not be further discussed; useful references^15^, ^16^ and a testing guide^17^ are available.

Although not specified by the item descriptor, including Fragile X testing (FRAXA) and urine metabolic screen in first tier testing may be warranted, given that FRAXA accounts for 2% of non‐syndromic ID^18^, ^19^ and metabolic conditions underlie 0.8–1.8% of GDD, some of which are treatable.^19^, ^20^, ^21^ CMA, FRAXA and urine metabolic screen together provide a diagnosis for up to 15–20% of GDD/ID.^22^

#### Consideration of patient perspectives

It is essential to ensure that the clinician's and family's motivations for testing are aligned. A genetic diagnosis may have clinical utility: guiding management, avoiding invasive investigations and ending the diagnostic odyssey. Cost‐effectiveness and increased sensitivity when compared to sequential standard testing has also been proven.^23^, ^24^ For the patient and their families, it may provide closure, enable easier access to services, facilitate reproductive options and allow interaction with an empathetic community via patient support/advocacy groups.

However, the psychosocial implications for a family in attaining a genetic diagnosis cannot be underestimated. A genetic diagnosis may represent a further loss of hope for families already caring for a child with significant medical and intellectual issues, given that such conditions are generally life‐long and in most neurodevelopmental disorders, leave little hope for cure. Clinically, rare genetic diagnoses often have scant natural history information. Genomic data filtering is still evolving; and therefore families may receive unclear or unexpected results that create uncertainty and anxiety, and this needs to be discussed carefully as part of the consent process. For some communities, there is guilt and cultural stigma associated with a genetic diagnosis.^25^, ^26^ Further, the fear of uncovering unexpected family relationships such as non‐paternity may deter some families. The insurance implications of a genetic diagnosis may also be a consideration regarding concerns around privacy of clinical and laboratory databases.

### Organising genomic testing

#### Consent

The above are important considerations for the paediatrician and family during the consent process, which is the first step in organising a genomic test. Consent is mandatory. Ethically, it applies to personal autonomy and self‐determination. Moreover, Australian laboratories are required to ensure that written informed consent has been obtained prior to proceeding with GT. These consent forms may be accessed via local clinical genetic services, which are listed on the CGE website.^27^ At the time of writing, a National Consent template is in development under the National Implementation Plan for the National Health Genomics Policy Framework, for adoption and adaptation across all Australian jurisdictions.

Informed consent requires a relevant summary of genetics for patients and explanation of possible outcomes to help manage patient/parent expectations. The main possible outcomes of testing can form the basis of the consent discussion (Fig. 1, Table 1). Printable resources for patients explaining genomics are available online.^35^, ^36^, ^37^

**Table 1:** Types of genomic testing and possible outcomes of testing

Term	Explanation	Additional considerations
Types of genomic tests		
Whole genome Sequencing (WGS)	WGS utilises next‐generation sequencing (NGS) to sequence the entire genome including the exons, introns and intergenic regions, and even the mitochondrial genome	This has the highest yield, but also generates the most data for analysis. It also allows for accurate copy number (deletion/duplication) analysis. To maximise diagnostic yield and assist interpretation of VUS, a trio WES or WGS is highly recommended and should be considered first rather than singleton testing
Whole exome sequencing (WES)	WES utilises NGS to sequence all coding regions of genes. This does not include the intergenic regions or deep introns and may not include the mitochondrial genome	See above considerations WES sequences only the exons, or protein coding regions of the genome, as well as the immediately adjacent intronic sequence in which variants affecting mRNA splicing may be identified. This includes approximately 50 million base pairs of DNA or ~2.8% of the genome
Gene panel	A particular predefined subset of genes is analysed, either in its own genomic test or as a part of WES or WGS	It may be appropriate to only examine the specific genes related to the clinical presentation. For example, in Noonan syndrome, there are approximately 20 causative genes reported to date. Therefore, examination only of those genes that are associated with Noonan syndrome is undertaken
Single gene sequencing	Conventional Sanger sequencing of a single gene	When the diagnosis is both clinically and genetically homogenous (e.g. Cystic Fibrosis and the *CFTR* gene), sequencing the single causative gene may be undertaken. Whilst WES/WGS and tests are covered by the new Medicare item number, to date most single gene tests are non‐rebatable and should be discussed with or referred to the local genetics services
Possible outcomes of genomic testing		
Genetic cause identified	A likely pathogenic or known pathogenic variant in a disease gene associated with the subject's phenotype has been found	A genetic cause is identified in 29–57% of cases^7^, ^8^ and may provide additional information on the patient's condition, family recurrence, and, possibly, management and future prognosis. Additional information may be required to help inform the clinician and family, and local genetics services can assist with this
Variant of uncertain significance (VUS)	These are seen in up to 20–25% of cases^28^, ^29^ and may cause confusion and anxiety. A VUS result may be returned when there is insufficient evidence that an identified variant is the cause of the patient's condition	A VUS should not be used in clinical decision making, and may need further discussion with local genetics services, the laboratory, and even further research and time to clarify. Parental studies may be helpful to determine if a VUS is benign or pathogenic, and this is why doing a trio analysis upfront is so helpful. If the variant is inherited from an unaffected parent it may be considered less likely to be causative. Guidance on setting expectations including possibility of VUS results is provided on the CGE website^30^
Incidental finding (IF)	A finding that is unrelated to the initial indication of testing, but is of possible clinical importance. Examples include a cancer predisposition gene, or unrelated genetic condition such as Cystic Fibrosis carrier status. Mathematical modelling estimates their frequency to be 1.5–6.5%,^31^ while studies suggest that they are seen in 1–2% of tests conducted internationally^32^, ^33^	The implications associated with finding Ifs may be concerning for the patient or to the parents themselves. However, it is vital to highlight that most diagnostic genomic analysis is patient‐specific and phenotype‐focused, and therefore this approach will largely mitigate the risk. The identification and reporting of Ifs is a controversial area and raises additional issues such as insurance and screening. The Australian approach to Ifs differs from that in the USA where the American College of Medical Genetics recommends screening a certain set of ‘medically actionable’ genes (termed secondary findings) and recommends reporting of all Ifs.^32^ Such a practice facilitates surveillance or treatment in a person's lifetime. However, this approach is controversial and currently not standard practice within Australian genomics laboratories
Negative result	No causative variant is found. Possible explanations are: The underlying cause is not monogenic; it may be oligogenic or polygenic; the former is hypothesised to be the case in some patients with autism, explaining the lower diagnostic rate^9^, ^10^, ^11^, ^12^ The causative variant is in an as yet undiscovered gene The variant is not detected due to technical limitations of the test The condition is not genetic	Given gene discovery is dynamically occurring, with 300 novel genes identified per year,^34^ re‐examination of genomic data in 1–2 years will have increased yield. It is also important to consider additional causes of genetic conditions that will not be diagnosed on standard genomic testing, such as mitochondrial variants, methylation/ epigenetic alterations, deep intronic variants, repeat expansion disorders, and cryptic copy number variants. These can be discussed with local genetics services

Apart from potentially predictive and/or unwanted health information being inadvertently disclosed, the possible impact of informative GT results on insurance must be noted to families. A 5‐year moratorium was implemented by the Australian Financial Services Council regarding use of genetic data to determine insurance premiums^6^, ^38^; however, some insurers may require relevant GT results be disclosed with *new* applications for mortgage/income protection/life insurance.^6^ It is important to reassure parents that GT results have no impact on obtaining health insurance in Australia, or on insurance policies already in place. More information is available online.^38^

## Discussion with a Clinical Geneticist

Ordering GT under Medicare item 73 358 requires discussion with a clinical geneticist. The exact format of this is not specifically defined, and therefore discussion with the local genetics service is suggested, as each genetics unit will have developed their own protocol to facilitate testing. Written evidence of appropriate consultation should be submitted to the laboratory with the test request and patient consent forms, to ensure claims are not rejected because they do not meet the requirements of the Medicare Item number.

### Which test to order

Interrogation of a patient's genome can be undertaken via various approaches, including WGS, WES, gene panel and single gene sequencing. Choosing the appropriate test requires an understanding of the types of GT (Table 1).

ID, severe epilepsy and some MCA presentations are genetically and phenotypically heterogeneous, that is the clinical presentation may be associated with any one of potentially hundreds of genes. For childhood syndromes or ID/GDD, WES or WGS are the tests of choice as they offer a broad, agnostic screen. At the time this report was written, WES is more widely available and is less expensive than WGS. This will evolve in the future as WGS becomes more logistically and financially accessible.

When ordering WES or WGS, whether to test the patient in isolation (singleton testing; MBS item number 73358) or along with both biological parents (trio testing; MBS item number 73359) is an important consideration. The latter (trio) approach is *highly* recommended given it simplifies analysis. Parental sequencing is used to triage inherited variants of interest in the sequencing data, therefore improving laboratory reporting efficiency and diagnostic yield.^39^, ^40^ It is also a more streamlined clinical test as trio testing identifies fewer variants of uncertain significance (VUS; Table 1) than singleton testing. However, in some instances only one parent is available for testing, or parents may have reservations about genomic sequencing of their own data because of privacy or insurance implications and a singleton may be undertaken.

When organising a trio‐based genomic test, consent must be obtained from both biological parents and for the child. A cautious and inclusive approach needs to be undertaken where parents are separated or divorced, and discussion with both parents is recommended.

Most laboratories will require DNA to be extracted from a blood sample (4–10 mL EDTA), while some laboratories will also offer GT with DNA extracted from buccal swabs or saliva samples. Discussion with the testing laboratory and local genetics services is recommended.

### Choice and prerequisites of laboratory

Testing must be done by a National Association of Testing Authorities (NATA)‐accredited Australian laboratory, and the choice of laboratory will often be determined by location and availability. Discussion with the local genetics unit may be required. Testing by international laboratories is not funded through Medicare.

Most Australian states have local institutional molecular laboratories, which will have specific requirements and documentation that must be fulfilled prior to ordering GT (Fig. 1). Many will require a copy of the consent: one for the patient and one each from the parents if a trio WES/WGS is being requested. In addition, detailed clinical or phenotypic information is critical to genomic analyses. Although a national referral form is being developed, at present each laboratory will have its own specific phenotype form and often a separate laboratory‐specific request form may also be needed for the patient. Relevant family history and the phenotypic details of the patient should be clearly summarised, as this will inform the genomic analysis. Occasionally, a parent has a similar phenotype to the patient, and this needs to be taken into account when variants are analysed. It is essential to provide such information to the laboratory at the time of ordering. The use of standardised nomenclature such as Human Phenotype Ontology terms^41^ is recommended when detailing the phenotype as these are utilised to examine genomic data. Human Phenotype Ontology is a dynamically curated resource of standardised phenotypic terminology that provides a useful interface between the disciplines of genomic and clinical medicine, thus enabling tailored data interrogation.

### Return of results

GT is often complex and results may take weeks to months depending on local laboratory availability, though expedited options are available on request in some laboratories. As GT is integrated into mainstream paediatric practice, general paediatricians will become more comfortable with explaining possible results (Fig. 1 and Table 1). However, the conditions being diagnosed are often rare, and VUS or incidental findings (IFs) may be challenging to communicate to families (Table 1).^42^ Ideally, results disclosure should have a nuanced, tailored approach and may be dependent on the family's and clinician's genomic literacy. If pretest counselling as part of the consent process has been comprehensive, the family usually has a relatively good understanding of the genomic process and can grasp the complex concepts of genomic results.^42^ Printable fact sheets for patients explaining VUS, IF and uninformative results are available.^43^

A confirmed genetic diagnosis may be straightforward with easy access to medical information regarding natural history, co‐morbidities and therefore management. More often, given the genetic heterogeneity of ID, the causative gene is uncommon and further information for both the clinician and family may be obtained through the local clinical genetics unit or via the referenced resources.^43^ If a trio test has been undertaken, recurrence risk may also be clarified. If a singleton test was conducted and a parent or sibling is also suspected to be affected, a Medicare rebate is available to test for the causative variant in nuclear family members (Items 73361, 73362). This may require pre and posttest guidance from the local genetics services.

In the event of a VUS, consultation with clinical genetics is helpful, as further detailed phenotyping or functional studies may be required to clarify whether the change in the specific gene fits with the clinical presentation reported in the literature. In explaining a negative or rather an uninformative result, it is important to highlight to families that regular re‐analysis of data can provide an additional diagnostic yield of 10–15%, due to the ongoing discovery of new gene‐phenotype relationships.^23^, ^44^ This is recommended every 18 months,^44^ has been assigned a Medicare item number (Item 73360), and is available twice after the initial test. This should also be discussed with the local genetics services, especially if there are mitigating circumstances that warrant a faster reanalysis (e.g. in the event of a pregnancy or progressive disease). Some conditions are not detected by WES; these include balanced disease‐causing rearrangements, mitochondrial variants, methylation abnormalities (as underlies Angelman and Prader‐Willi syndromes) and small structural variants such as deletions/duplications; the latter are below the resolution of CMA but could potentially be diagnosed by WGS.

## Conclusion

GT will increasingly be integrated into mainstream paediatrics, and a suggested pathway to streamline the process for any child whose clinical features meet the Medicare requirements is outlined in Figure 1. However, complex issues may arise, including consent matters, logistics of ordering testing, result interpretation and expediting testing in the event of a pregnancy or a management concern. There may also be a number of potential sources of uncertainty for the family arising from GT.^45^ The involvement of the genetic counsellor has been proven to be associated with better patient‐reported outcomes.^34^, ^46^, ^47^ ‘Embedding’ a genetic counsellor within a specialty that routinely uses GT is a model of care that is being evaluated. Access to local genetics service is recommended at any time during the process and an expanding list of resources is being developed to help clinicians and families understand, implement and streamline GT, both now and in the future.^43^
